# Influence of Dental Glass Fibers and Orthopedic Mesh on the Failure Loads of Polymethyl Methacrylate Denture Base Resin

**DOI:** 10.3390/polym13162793

**Published:** 2021-08-20

**Authors:** Muhammad H. Rana, Sharaz Shaik, Mohammad S. Hameed, Samar Al-Saleh, Eman M. AlHamdan, Abdullah Alshahrani, Abdulaziz Alqahtani, Ahmed Heji Albaqawi, Fahim Vohra, Tariq Abduljabbar

**Affiliations:** 1 Department of Prosthetic Dentistry, College of Dentistry, King Khalid University, Abha 61421, Saudi Arabia; haseebrana80@gmail.com; 2 Department of Prosthetic Dentistry, Lenora Institute of Dental Sciences, Rajahmundry 533101, India; sharazshaik@gmail.com; 3 Department of Diagnostic Sciences and Oral Biology, College of Dentistry, King Khalid University, Abha 61421, Saudi Arabia; mohammad.shahul@gmail.com; 4 Department of Prosthetic Dental Sciences, College of Dentistry, King Saud University, Riyadh 11545, Saudi Arabia; Salsaleh@ksu.edu.sa (S.A.-S.); ealhamdan@ksu.edu.sa (E.M.A.); asalshahrani@ksu.edu.sa (A.A.); absalqahtani@ksu.edu.sa (A.A.); fvohra@ksu.edu.sa (F.V.); 5 Department of Restorative Dental Sciences, College of Dentistry, University of Hail, Hail 55476, Saudi Arabia; a.albaqawi@uoh.edu.sa; 6 Eng. Abdullah Bugshan Research Chair for Dental and Oral Rehabilitation, College of Dentistry, King Saud University, Riyadh 11545, Saudi Arabia

**Keywords:** dental fiberglass framework, orthopedic casting tape, PMMA acrylic denture

## Abstract

The aim of the present study was to evaluate the fracture loads of polymethyl methacrylate (PMMA) complete denture bases reinforced with glass-fiber mesh and orthopedic casting tape (OCT) in comparison to conventional PMMA dentures under artificial aging. Dental fiberglass framework (Group 1) and OCT (Group 2 and 3) reinforced PMMA acrylic dentures were fabricated on the edentulous ridge. Ten PMMA dentures without reinforcement (Group 4) were included as controls. All specimens were placed in a chewing simulator chamber, and fatigue load was applied. To assess the fracture loads, static loads with a universal testing machine were applied. Fractured specimens in each group were evaluated under a scanning electron microscope. The data were statistically analyzed employing analysis of variance and Tukey post-hoc test. The association of denture weight and thickness on fracture load was assessed using Pearson and Spearman correlations. Dental fiberglass (Group 1) displayed the highest fracture load (692.33 ± 751.41 N), and Group 4 (control) exhibited the lowest fracture loads (281.41 ± 302.51 N). Dentures reinforced with fiberglass mesh framework exhibited intact fractures. In contrast, Group 2 and 3 specimens using OCT demonstrated ditching fractures. It was observed that the thickness and weight of all the reinforced specimens influenced the load required to fracture the dentures (*p* < 0.001). Denture specimens strengthened with OCT (Groups 2 and 3) exhibited failure loads lower than dental fiberglass (Group 1) specimens but higher than unreinforced controls.

## 1. Introduction

Heat and auto-polymerized polymethyl methacrylate (PMMA) acrylic resin are the most widely used material in the field of dentistry for prosthodontic and orthodontic appliances [1]. They are employed in a variety of restorations, i.e., complete, and partial dentures, provisional restorations or surgical aesthetic correction [2]. Excellent and pleasing esthetics, low water sorption, biocompatibility, ability to repair, manipulation ease and simple processing technique are some of the advantages of PMMA material that contribute to its success in clinical dentistry [3]. However, PMMA exhibits a compromise in mechanical and physical properties, i.e., low flexural strength (FS), low impact strength (IS) and low surface hardness, which leads to reduced clinical longevity of the prosthesis along with patient dissatisfaction [4]. The most common problem faced by the patients and dentist, associated with the PMMA denture bases, are fractures [5]. Johnston et al., revealed that nearly 68% of acrylic denture bases are fractured after a few years of fabrication, predominantly due to impact failure [6].

Forces usually responsible for resin denture prosthesis failures are flexural fatigue and impact force [7]. In order to overcome the mechanical shortcomings of PMMA dentures, different reinforcement methods have been adopted to improve their fatigue and fracture loads [8]. Initially, trials were conducted on the incorporation of metal wires and cast metal plates into PMMA acrylic resins [9,10]. However, a weak bond between the metal wires and acrylic resin was reported, resulting in poor mechanical properties of denture prosthesis [11]. In addition, incorporated metal plates undergo corrosion and form corrosion products causing decreased strength and potential staining of the denture [12]. Interestingly, the addition of organic and inorganic fibers to acrylic denture bases is also employed for resin reinforcement. Different fibers include Metal, Kevlar^®^, glass, sapphire, polyester, carbon graphite and rigid polyethylene [13]. The incorporation of fibers in acrylic dentures improves the mechanical properties, i.e., transverse, tensile and impact strengths. Moreover, fiber reinforcement provides better esthetics and improved bonding to the resin when compared to other methods opted for reinforcement [14].

Among the different types of fibers used, glass fibers (GF) have gained much attention in the field of dentistry [15]. They are more esthetically pleasing, flexible and biocompatible when compared to other types of fibers, i.e., aramid carbon/graphite fibers that display poor aesthetics and weak bond with the acrylic resins [16]. Moreover, dentures using GF reinforcement are beneficial to the dental technician, dentist, and the patient, as they are fabricated in a short period of time and weigh less compared to the conventionally employed metal reinforcements. John et al., reported that glass, aramid or nylon fibers were effective in increasing the flexural strength of denture resins [17]. They also reported that among these fibers, glass-fiber-reinforced specimens, in particular, displayed the highest flexural strength [17]. Recently a few clinical studies have been conducted using glass fiber mesh as denture reinforcement for mechanical improvements. Fiber mesh is composed of e-glass fiber, and it is claimed that a denture reinforced with SES Fiber Mesh is three times stronger than a conventional denture without any reinforcement [18]. However, data related to the fracture load after reinforcing acrylic dentures with the glass fiber mesh is limited. Similarly, orthopedic casting tape (OCT) has been used for fracture management and ensures excellent outcomes of immobilization and rigid support in the field of orthopedics. However, their role in denture reinforcement has not been investigated.

From the available indexed literature, it was observed that data related to mechanical properties of complete dentures reinforced with glass-fiber mesh is inadequate. Moreover, the use of OCT for complete denture reinforcement is a novel concept in the reinforcement of PMMA acrylic resin denture bases. Therefore, it is hypothesized that PMMA acrylic resin denture bases reinforced with glass-fiber mesh and OCT will display better fracture loads when compared with the PMMA dentures bases without reinforcement. Thus, the aim of the present study was to evaluate the fracture strength of PMMA complete acrylic denture bases reinforced with glass-fiber mesh and OCT in comparison to conventional PMMA complete denture bases under artificial aging.

## 2. Materials and Methods

This study was submitted, reviewed, and approved by the center for specialist dentistry and research (UDRC/010-20). The ethical standards of the 1964 Helsinki declaration and national and/or institutional research committee were strictly followed while performing all the procedures. Forty PMMA stone-cast edentulous-standard denture base plates were prepared. Ten samples were prepared for each reinforcement of PMMA: acrylic with dental fiberglass framework (Group 1: SES Mesh), orthopedic cast tape (Group 2: Delta Lite Plus) and second orthopedic cast tape (Group 3: orthopedic casting tape). Ten samples were fabricated without reinforcing mesh using conventional heat-cured acrylic; these served as controls (Group 4). The study group details are presented in Table 1.

### 2.1. Master Cast Preparation

Forty edentulous maxillary models were fabricated with type 3 dental stone (Durguix, Protechno, Spain.) using an edentulous silicon mold (EDE1001-UL-MO, Nissin Dental Products Inc., Kyoto, Japan). Model surface discrepancies were removed.

### 2.2. Acrylic Denture Base Fabrication

All prepared stone casts were treated with two layers of separating medium (Cold molsem d seal- Aqua seal, India) prior to waxing up for denture fabrication. For the wax-up of acrylic resin denture samples, in each group, two layers of 1.0 mm thick wax sheets (Preparation wax, BEGO Bremer Goldschlägerei Wilh. Herbst GmbH & Co. KG, Frankfurt, Germany) was adapted onto the functional cast area. Stops 2 mm wide and 5 mm long were created by removing wax in the canine, first molar regions bilaterally and in the mid-palatal region. The stops were filled with flowable resin composite (Premise flowable composite (Kerr, Corp, Orange, CA, USA) and light-cured with a dental light-curing unit (SES Curing Unit, Inno dental Co. Ltd., Seoul, Korea). The reinforcing mesh in respective study groups was adapted over the entire wax sheet, 2 mm short of the vestibule and posterior palatal seal area (posterior border). The mesh was adapted into the cutout wax tops to secure them. The adaptations for all mesh reinforcements were performed with a vacuum apparatus (SES vacuum unit, Inno dental Co. Ltd., Seoul, Korea). The mesh in Group 1 specimens was light polymerized using a standard curing system (SES Curing Unit, Inno dental Co. Ltd., Seoul, Korea). However, the OCT among Group 2 and Group 3 specimens was water sprayed and adapted manually, followed by vacuum adaptation. The casting tapes (Groups 2 and 3) were auto-polymerized for 8 h. The reinforcing mesh and casting tapes among Groups 1, 2 and 3 were secured to the resin stops with cyanoacrylate, preventing the displacement of the mesh during acrylic resin dough packing in denture processing. The reinforcement meshes were covered with two sheets of 1.0 mm preparation wax, followed by adaptation among all groups. Among Group 4 specimens (controls), no reinforcement mesh was placed, and two sheets of 1.0 mm preparation wax were applied and adapted.

### 2.3. Processing of Denture Plates

**Figure 1:**
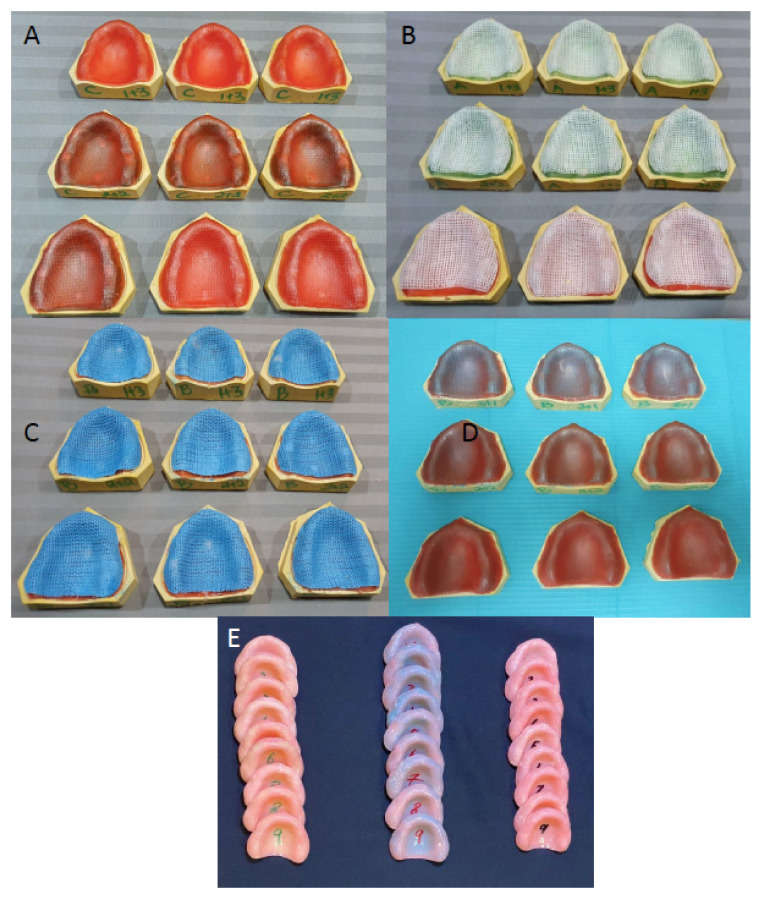
PMMA resin reinforcement mesh groups. Group 1. Dental fiberglass framework reinforcement (**A**). Group 2. Orthopedic casting tape, Delta Lite Plus reinforcement (**B**). Group 3. Orthopedic casting tape, Shanghai Nineluck reinforcement (**C**). Wax up of all reinforced groups (**D**). Processed dentures (**E**).

All the waxed-up specimens were developed with dental stone within the dental flask. After the complete setting of stone plaster, flasks were dewaxed. The wax left on the stone surface after dewaxing was flushed away with the help of boiling water. The mold space obtained after dewaxing was then used to fabricate the test specimens. The molds were left open, air-dried and cooled at room temperature. A separating agent (cold mold seal; Dental products of India, DPI) was smeared on the surface and dried. Heat cure acrylic was mixed following the manufacturer’s guidelines and packed in the flasks at the dough stage. All the flasks were placed in the hydraulic press (Sirio Dental, Meldola, Italy), and the entire mold and clamp assembly was placed in the curing unit (Wassermann Dental-Maschinen GmbH, Hamburg, Germany), maintaining 72 °C temperature for almost 90 min and 95 °C for 30 min. Prior to deflasking, the flasks were cooled at room temperature for 30 min to release the stresses. The dentures were recovered from the flask and finished with a series of silicon carbide papers (Buehler Ltd., Esslingen, Germany). The surface of the specimens was polished and smoothened using a lapping machine (MetaServ 250, Buehler Ltd., Esslingen, Germany). Figure 1 shows the mesh applications and processed PMMA resin denture plates. The weight (gm) and thickness (mm) of each denture were measured after finishing and polishing.

### 2.4. Fatigue Load Application

All specimens were artificially aged by thermocycling for 50,000 cycles between 5 °C and 55 °C water baths at a dwelling time of 10 s. All specimens were placed in a chewing simulator chamber and exposed to 10,000 cycles at 20 N load in distilled water. The opposing loading surface was in the form of acrylic resin balls of 15 mm diameter (Meliodent Heat Cure, Kulzer GmbH, Frankfurt, Germany) [19], contacting the anterior palatal slopes.

### 2.5. Fracture Testing

**Figure 2:**
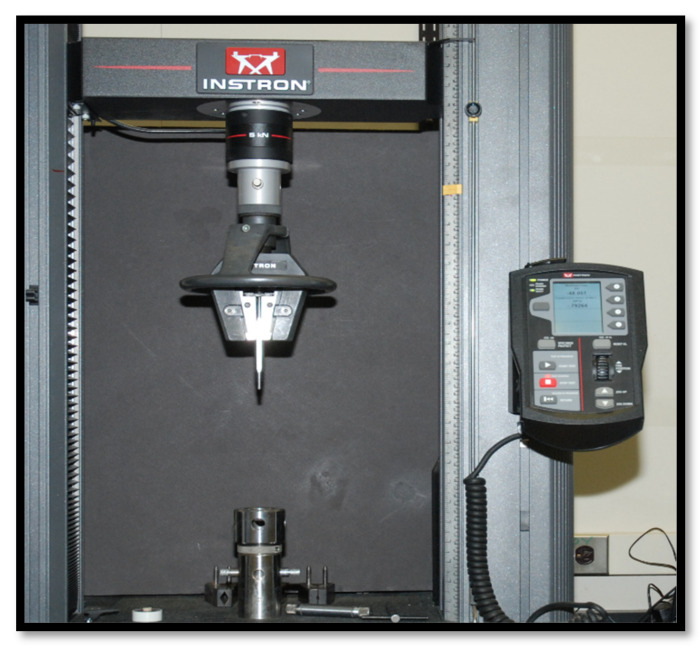
Testing equipment (Instron testing machine) used for denture fractures.

All denture plates were assessed for fracture loads in Newton (N). The denture plates were secured on a customized metal stage and a controlled continuous load was applied on the anterior palatal slope of the plate with a customized half-round (10 mm diameter) metal probe. Static compressive loads were applied at a crosshead speed of 1mm/min. The fracture testing setup is shown in Figure 2.

Four fractured specimens in each group were evaluated under scanning electron microscopy (SEM) (JSM-6513, JEOL, Tokyo, Japan). The fractured resin surface was prepared by placement on aluminum stubs and sputter coating with gold for 2 min (Baltec SCD sputter, Scotia, NY, USA). The assessments and evaluations were made at an accelerating voltage of 30 kV utilizing SEM. SEM micrographs of the specimens were obtained at multiple magnifications for qualitative assessment of the fracture surfaces showing the reinforcing mesh and casting tapes. The data were statistically analyzed by statistical software (Version 20, SPSS Inc., Chicago, IL, USA) employing analysis of variance (ANOVA) and Tukey post-hoc multiple comparison test. The association of denture weight and thickness to fracture loads was assessed using Pearson and Spearman correlations.

## 3. Results

The fracture loads of PMMA acrylic resin plates are presented in Table 2. Group 1 (dental fiberglass framework) displayed the highest fracture loads (692.33 ± 75.41N), and Group 4 (control) exhibited the lowest fracture loads (281.41 ± 30.51 N). ANOVA revealed a statistically significant difference in fractural load outcome among the different tested groups (*p* < 0.001). Furthermore, individual group comparison established that Group 1 displayed higher fracture loads than Group 2 (Orthopedic casting tape, Delta Lite Plus, BSN medical GMBH, Frankfurt, Germany) (487.40 ± 51.72 N), Group 3 (Orthopedic casting tape, Shanghai Nineluck Co. Ltd., Shanghai, China) (486.94 ± 52.39 N) and Group 4 specimens. Specimens in Groups 2 and 3 exhibited fracture loads higher than control specimens (*p* < 0.05) and comparable among themselves (*p* > 0.05).

**Figure 3:**
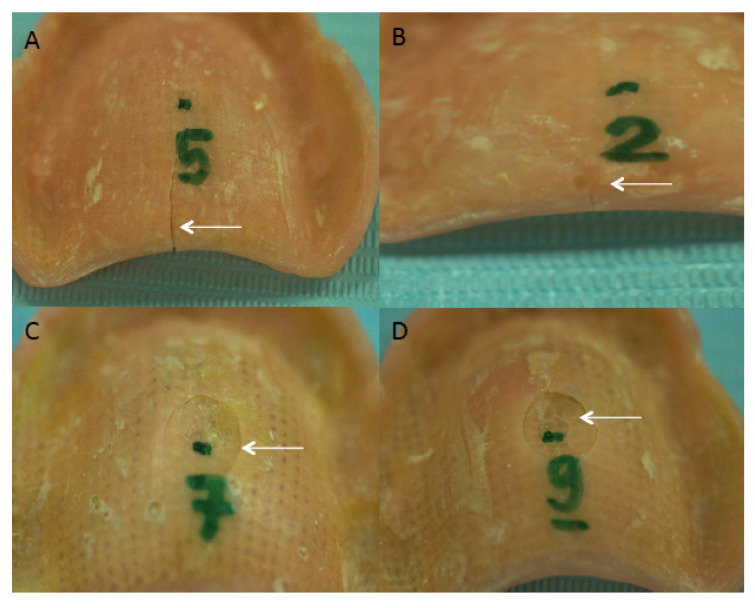
Fracture patterns of the PMMA resin plate samples in the study groups. (**A**,**B**), Showing straight line intact fracture patterns in Group 1 specimens. (**C**,**D**), Fractured specimens showing ditched fractures in specimens of Group 2 and Group 3, respectively.

The fractured specimens of reinforced groups are presented in Figure 3. The images showed that dentures reinforced with fiberglass mesh framework (group 1) exhibited intact failures (Figure 3A,B). Whereas Group 2 and 3 specimens, using OCT, demonstrated ditching fractures (Figure 3C,D). The correlation between thickness and weight of reinforced and non-reinforced acrylic dentures with the failure loads among the different experimental groups is presented in Table 3. It was found that the thickness and weight of all the reinforced specimens influenced the load required to fracture the dentures (*p* < 0.05).

**Figure 4:**
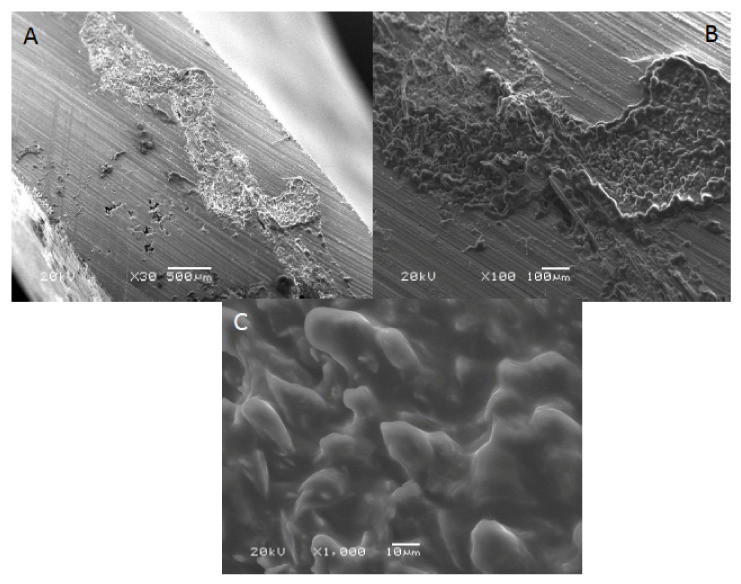
SEM images of acrylic denture reinforced with dental fiberglass framework (group 1) at (**A**) ×30, (**B**) ×100 and (**C**) ×1000 magnification.

**Figure 5:**
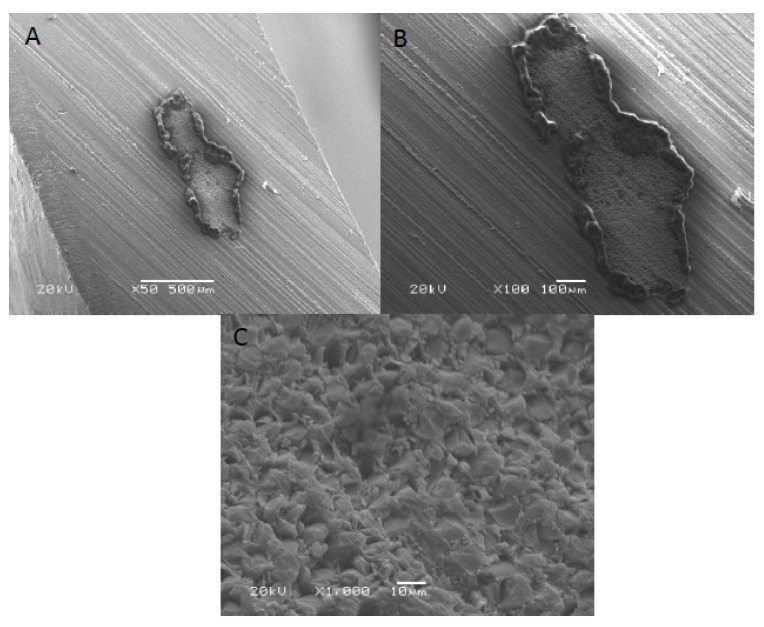
SEM images of OCT fibers (group 2) at (**A**) ×50, (**B**) ×100 and (**C**) ×1000 magnification.

**Figure 6:**
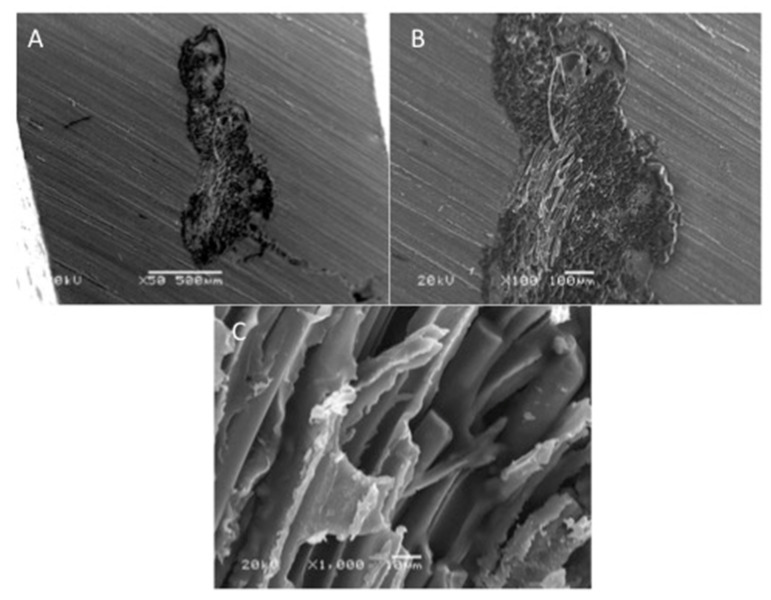
SEM images of OCT fibers (group 3) at (**A**) ×30, (**B**) ×100 and (**C**) ×1000 magnification.

SEM images of acrylic denture reinforced with Dental fiberglass framework are presented in Figure 4. A bundle of reinforcing fibers was observed at 30× magnification, with an approximate length of 2.5 mm (Figure 4A). In addition, flat and irregular fiberglass bundle fibers demonstrating rounded edges were observed (×100) (Figure 4B). Figure 3C presented a high magnification image of dental fiberglass unidirectional glass fibers embedded in a resin matrix (×1000) (Figure 4C). Figure 5 presents SEM images of OCT fibers observed at different magnifications. A bundle of fibers was observed with elevated margins, measuring approximately 500 × 200 um (Figure 5A,B). A honeycomb-pattern bundle of glass fibers with closely staked fibrils and comparatively few empty spaces around the individual fibers was appreciated (×100) (Figure 5C). Figure 6 presents SEM images of OCT used in Group 3 at magnifications of ×30, ×100 and ×1000. A bundle of fibers with raised boundaries having an estimated length of 1500 um was detected (Figure 6A,B). At higher magnification, individual elongated and parallel micro-fibers with scant matrices were observed (×1000). Individual fibrils were densely packed, flat and interconnected (Figure 6C).

## 4. Discussion

The present in vitro study was based on the hypothesis that complete acrylic dentures reinforced with a glass-fiber mesh and OCT display better fracture and failure loads when compared to dentures without any reinforcement (control) in a model simulating oral conditions. The use of glass fiber and OCT showed a significant improvement in the failure loads of PMMA resin denture plates compared to controls; therefore the hypothesis was accepted.

A strong denture base is required for clinical longevity, biologically acceptability and aesthetically pleasing outcomes [15,20]. The literature revealed that an adult human with an intact dentition usually exerts a 300 to 700 N dynamic masticatory load [21,22]. The force required to fracture non reinforced denture is around 706 N, while reinforced denture bases required 903 N of masticatory force, which is much greater than the force required to fracture the denture [17,23]. Moreover, it was also found that the oral environment influences the threshold for denture fractures intraorally. Therefore the study employed the use of thermocycling and chewing simulation to closely simulate the oral environment for testing failure loads of experimental denture plates [24].

The literature revealed that fracture loads of reinforced acrylic denture prostheses are dependent on the mechanical properties of the material used [25,26]. Studies have suggested that glass fibers efficiently influence the mechanical strength of the acrylic denture bases [15,27]. However, certain factors, i.e., fiber type, diameter and length of the fiber, fiber resin ratio, orientation and location of the fiber, affect the reinforcement properties of different fibers used [28]. The dental fiberglass mesh and OCT used in the present study is a combination of strong glass fibers, substrate and resin [29]. In the present study, the dental fiberglass framework (group 1) demonstrated the highest fracture loads (692.33 ± 75.41MPa). Group 4 specimens with no reinforcement displayed the lowest fracture loads (281.41 ± 30.51 MPa) when compared to all the experimental groups.

In the present study, it was found that both OCT and fiberglass mesh are effective in strengthening the acrylic denture bases; this is due to the presence of glass fibers in the resin matrix. This is in line with the outcomes of the study conducted by Yu et al. [29]. However, differences in the flexural strength among reinforced investigated groups may be due to the differences in the distribution of glass fibers and their adhesion with the heat-activated acrylic resin [30]. Moreover, it can be assumed that different fiber orientations in tested groups may also be responsible for the difference in fracture loads of reinforced dentures [31]. It is also suggested that the most preferred OCTs consist of a minimum of 50% to 70% of filler content [32]. In addition, the authors predicted that the difference in fracture load among the two OCT groups might be due to the difference in fiber concentration.

In the present study, it was observed that the weight and thickness of the PMMA resin denture plates influence their fracture loads. This is in line with the outcomes of previous studies, which reported that 57% to 64% of acrylic resin denture fractures occur at the site of the least thickness of specimen [33,34]. Since biomechanical stresses are focused on these areas, it can be concluded that the increased thickness and weight of acrylic dentures affect the mechanical properties of dentures [35].

SEM analysis revealed detectable variations among the matrix and fiber morphology and configuration among materials. Material morphology was influenced by the orientation and concentration of fibers used. It was found that the dental fiberglass in Group 1 specimens showed a greater resin matrix with longer and larger fibers when compared to Groups 2 and 3 OCT reinforced PMMA dentures, justifying the greater flexural load in this group [13]. Similarly, it was also found that specimens in Group 1 showed intact fractures compared to Groups 2 and 3 specimens, which presented ditching fractures, thus confirming the higher fracture loads of Group 1 specimens.

The present study suggests the incorporation of orthopedic casting tapes and dental fiberglass to improve the fracture loads of PMMA acrylic dentures. However, these findings should be considered in light of the fact that the present study was in vitro, palatal load application in contrast to neutral zone load application was used and lateral loading was not performed. In addition, the impact of colored glass fibers on the aesthetic appearance of the prosthesis was not considered in the present study. Therefore, further long-term randomized controlled trials to assess the performance of PMMA resin-complete dentures reinforced with orthopedic casting tape and dental fiberglass are warranted.

## 5. Conclusions

Dental fiberglass reinforced PMMA dentures (Group 1) displayed the highest fracture loads among the study groups. Dentures without any fiber reinforcement (Group 4) exhibited the lowest fracture load. Denture specimens strengthened with OCT (Groups 2 and 3) exhibited failure loads lower than dental fiberglass (Group 1) specimens but higher than unreinforced controls. The thickness and weight of all the reinforced specimens influence the load required to fracture the dentures.

## Figures and Tables

**Table 1:** Materials used for acrylic reinforcement in study groups.

Study Groups	Materials for Reinforcement
Group 1	Dental fiberglass framework, Group Z: SES Mesh, Inno dental Co. Ltd., Seoul, Korea
Group 2	Orthopedic casting tape, Delta Lite Plus, BSN medical GMBH., Frankfurt, Germany
Group 3	Orthopedic casting tape 2, Shanghai Nineluck Co. Ltd., Beijing, China
Group 4 (control)	Heat Cure acrylic no reinforcements Meliodent Heat Cure, Kulzer GmbH., Frankfurt, Germany

**Table 2:** Means, SD and statistical comparison of fracture loads among study groups.

Group	Mean (N)	SD (N)	ANOVA *p*-Value	Kruskal-Wallis *p*-Value	Group Comparison	*Z*-Value	Adjusted *p*-Value
1	692.33	75.41	<0.001	<0.001	2 vs. 3	−2.27	0.03
					2 vs. 4	2.59	0.02
2	487.40	51.72			3 vs. 4	4.86	0.00
3	486.94	52.39			1 vs. 2	0.02	0.99
					1 vs. 3	−2.26	0.03
4	281.41	30.51			1 vs. 4	2.61	0.03

Group 1: Dental fiberglass framework, SES Mesh, Inno dental Co. Ltd., Korea. Group 2: Orthopedic casting tape, Delta Lite Plus, BSN medical GMBH, Germany. Group 3: Orthopedic casting tape, Shanghai Nineluck Co. Ltd., China. Group 4: Heat Cure acrylic with no reinforcements (control). *p*-value: significance set at α = 0.05. *Z*-value: standard score.

**Table 3:** Correlation of thickness and weight of the specimens with the failure loads among the groups.

Variable	Pearson CC	Pearson *p*-Value	Spearma CC	Spearman *p*-Value
Thickness	0.751	<0.001	0.822	<0.001
Weight	0.812	<0.001	0.862	<0.001

CC: correlation coefficient.

## Data Availability

The data is available on contact with the corresponding author.
